# Inhibition of AKT induces p53/SIRT6/PARP1-dependent parthanatos to suppress tumor growth

**DOI:** 10.1186/s12964-022-00897-1

**Published:** 2022-06-17

**Authors:** Yizheng Zhang, Chuchu Zhang, Jiehan Li, Meimei Jiang, Shuning Guo, Ge Yang, Lingling Zhang, Feng Wang, Shiqi Yi, Jiangang Wang, Yang Fu, Yingjie Zhang

**Affiliations:** 1 Department of Health Management, The Third Xiangya Hospital, Central South University, Changsha, China; 2 Department of Gastrointestinal Surgery, The First Affiliated Hospital of Zhengzhou University, Zhengzhou, 450052 China; 3 School of Biomedical Sciences, Hunan University, Changsha, 410082 China; 4 Department of Ophthalmology, The First Affiliated Hospital of Zhengzhou University, Zhengzhou, 450052 China; 5 Department of Laboratory Medicine, The Third Xiangya Hospital, Central South University, Changsha, 410013 China; 6 Department of Gastroenterology, The Tenth People’s Hospital of Shanghai, Tongji University, Shanghai, 200072 China; 7 Center for Reproductive Medicine, The First Affiliated Hospital of Zhengzhou University, Zhengzhou, 450052 China; 8 College of Biology, Hunan University, Changsha, 410082 China; 9 Department of Pathology and Neuropathology, University Hospital Tuebingen, 72076 Tuebingen, Germany

**Keywords:** AKT, p53, Parthanatos, Cell death, SIRT6, Colorectal cancer

## Abstract

**Background:**

Targeting AKT suppresses tumor growth through inducing apoptosis, however, during which whether other forms of cell death occurring is poorly understood.

**Methods:**

The effects of increasing PARP1 dependent cell death (parthanatos) induced by inhibiting AKT on cell proliferation were determined by CCK-8 assay, colony formation assay, Hoechst 33,258 staining and analysis of apoptotic cells by flow cytometry. For the detailed mechanisms during this process, Western blot analysis, qRT-PCR analysis, immunofluorescence and co-immunoprecipitation were performed. Moreover, the inhibition of tumor growth by inducing p53/SIRT6/PARP1-dependent parthanatos was further verified in the xenograft mouse model.

**Results:**

For the first time, we identified that inhibiting AKT triggered parthanatos, a new form of regulated cell death, leading to colon cancer growth suppression. For the mechanism investigation, we found that after pharmacological or genetic AKT inhibition, p53 interacted with SIRT6 and PARP1 directly to activate it, and promoted the formation of PAR polymer. Subsequently, PAR polymer transported to outer membrane of mitochondria and resulted in AIF releasing and translocating to nucleus thus promoting cell death. While, blocking PARP1 activity significantly rescued colon cancer from death. Furthermore, p53 deletion or mutation eliminated PAR polymer formation, AIF translocation, and PARP1 dependent cell death, which was promoted by overexpression of SIRT6. Meanwhile, reactive oxygen species production was elevated after inhibition of AKT, which might also play a role in the occurrence of parthanatos. In addition, inhibiting AKT initiated protective autophagy simultaneously, which advanced tumor survival and growth.

**Conclusion:**

Our findings demonstrated that AKT inhibition induced p53-SIRT6-PARP1 complex formation and the activation of parthanatos, which can be recognized as a novel potential therapeutic strategy for cancer.

**Video Abstract**

**Supplementary Information:**

The online version contains supplementary material available at 10.1186/s12964-022-00897-1.

## Background

Colorectal cancer (CRC) was the third most commonly diagnosed cancer and the second leading cause of cancer death worldwide estimated in 2018 [1]. Europe, Australia, North America and Eastern Asia are the regions with the highest CRC incidence rates. Recently, surgical resection combining with chemotherapy is the main strategy of CRC treatment and under these managements, the 5-year survival rate of early diagnosed localized CRC exceeds 80%. However, the 5-year survival rate decreases to < 10% for advanced stage (stage IV) CRC [2]. Resistance to apoptosis is found to make CRC cells survive under chemotherapy [3] which always leads to treatment failure. Thus, it is urgently needed to explore novel target therapy methods with specific mechanism in order to overcome apoptosis resistance in patients with advanced stage CRC.

Poly [ADP-Ribose] polymerase 1 (PARP1)-dependent cell death (parthanatos) is a novel form of non-apoptotic regulated cell death, which occurs without apoptotic body formation and small-size DNA fragmentation [4, 5]. Parthanatos is triggered by PARP1 hyperactivation, which results in poly(ADP-ribose) polymer (PAR polymer) synthesized, and apoptosis-inducing factor (AIF), after binding to PAR polymer, released from mitochondria into nucleus to produce parthanatotic chromatinolysis [6]. Parthanatos has been proved to be involved in a lot of diseases including macular degeneration [7], parkinson's disease [8], smoke-related lung diseases [9] and oxidative stress-related hearing disorders [10]. In addition, researches also showed that chemical induced parthanatos contributed to cell death in various types of neoplasm, such as melanoma [11, 12], glioma [13], esophageal cancer [14] and breast cancer [15], suggesting parthanatos induction could be a potential therapeutic strategy to overcome apoptosis resistance. Although studies revealed that parthanatos might associated with oxidative injury, depletion of NAD+ and ATP and mitochondrial inner transmembrane potential dissipation [16, 17]. However, which specific signal pathway and related mechanism that trigger PARP1 hyper-activation and parthanatos in cancer remains unknown.

AKT, a serine-threonine kinase, is also regarded as a master regulator of cellular survival and growth [18, 19]. Abnormal overexpression or activation of AKT was observed in various neoplasms [20], which also contributed to drug resistance and apoptosis blocking [21]. Therefore, inhibition of AKT has been researched as a potential therapeutic strategy to enhance drug sensitivity [22]. AKT overexpression suppresses the activation of the p53 signaling pathway via inducing the antiapoptotic Bcl-xL protein and enhancing MDM2, a negative regulator of p53, which has been regarded to play a critical role of apoptosis resistance and cellular survival [21, 23]. Previous studies revealed the crosstalk between p53 and PARP1, in which, activation of PARP1 requires the interaction of activated p53 while mutant or inactive p53 loses such function [24, 25]. Recently, MDM2-p53 pathway was revealed to involve in parthanatos during traumatic brain injury [26]. It is plausible to make a hypothesis that AKT activation triggers parthanatos via p53 pathway. Although blocking AKT signaling cascades to induce apoptotic cell death has been researched for many years in many neoplasms, including CRC [27]. However, the relationship between AKT inhibition and parthanatos remains unreported.

SIRT6 is a member of sirtuin family, which exhibits both NAD+-dependent deacetylase and ADP-ribose transferase enzyme activity [28, 29]. It plays critical roles in many biological processes like metabolism, DNA stability and repair, cell proliferation and differentiation [30, 31]. SIRT6 serves as a tumor suppressor and is observed decreased in various cancers such as colorectal, hepatocellular and pancreatic cancers [32–34]. SIRT6 shows a close relationship with cell death. Our previous study revealed that, AKT/FoxO3a pathway regulates SIRT6 expression in colon cancer and inhibition of AKT triggers SIRT6 transcription via FoxO3a activation, which significantly promotes cell death [35]. Meanwhile, SIRT6-p53 interaction was reported to regulate cardiolipin de novo biosynthesis [36]. Previous study also reported that SIRT6 could activate PARP1 through monoADP-ribosylation and the activated PARP1 modified HMGB1 by polyADP-ribosylation [37]. Therefore, we assume that SIRT6 may play an important role in AKT inhibition triggered parthanatos through p53-SIRT6-PARP1 complex formation.

Here, in this study, inhibition of AKT was found to induce parthanatos, triggering PARP1 hyper activation, PAR polymer accumulating and AIF translocation to nucleus. During this process, p53 interacted with PARP1 directly, which was indispensable for PARP1 activation and parthanatos. Mutant or absence of p53 dramatically abolished AKT inhibition triggered parthanatos in different cancer types. Protective autophagy was detected at the same time, which was beneficial for enhancing the viability of tumor cells. Our findings firstly discovered that AKT inhibition inducing not only apoptosis but also parthanatos and might partially explained the mechanism of AKT-p53-PARP1 axis. AKT related parthanatos can be regarded as a potential clinical therapeutic strategy in cancer treatment.

## Methods

### Cell culture and treatment

The human colon cancer cell lines, HCT116, HT29, SW480, SW620, the human embryonic kidney cell lines HEK293T, the human hepatocellular carcinoma cell lines HepG2 and the human prostate cancer cell lines DU145 were obtained from American Type Culture Collection (ATCC). Human colon cancer cell line with p53 knock out (HCT116 p53–/–) was generously provided by Dr. Bert Vogelstein (Johns Hopkins University, Baltimore, MD, USA). SW480, SW620, HT29 and DU145 were cultured in RPMI 1640, HEK293T, HepG2 were cultured in DMEM, HCT116 WT and HCT116 p53–/– were cultured in McCoy’s 5A modified media, supplemented with 10% fetal bovine serum (FBS), penicillin (100units/ml), and streptomycin (100 μg/ml) in 5% CO_2_ at 37 °C in a humidified incubator. All agents of SC66, TIC10 and 3AB diluted with DMSO were added in the medium directly before detection. In the aspect of transfection experiments, Lipofectamine 2000 transfection reagent was used following the supplier’s instructions. The medium was replaced with fresh culture medium after 5 h. Cells were then examined at 24–48 h after transfection.

### Antibodies and reagents

Primary antibodies against phospho-AKT (S473), pan-AKT, phospho-FoxO3a (S253), totalFoxO3a, phospho-p65 (S276), total-p65, cleaved-caspase3, cleaved-caspase1, cleaved-PARP1, LC3 and p62 were purchased from Cell Signaling Technology (Boston, Massachusetts, USA), total-caspase3, PAR and p-MLKL(S358) was purchased from abcam (Cambridge, UK), p53, PARP1, AIF, pan-AKT, totalFoxO3a, total-p65, GPX4 and β-actin were purchased from proteintech (Wuhan, Hubei, China), Cox IV, H3 and HRP conjugated anti-rabbit/mouse secondary antibodies were purchased from Abbkine (Wuhan, Hubei, China). Lipofectamine 2000 was purchased from Thermo Fisher Scientific (Waltham, Massachusetts, USA). AKT inhibitor SC66, TIC10, autophagy inhibitor CQ, ULK101, pan-caspase inhibitor Z-VAD-FMK and PARP1 inhibitor 3AB were purchased from Selleck (Houston, Texas, USA). CCK-8 kit was from 7 Sea Biotech (Shanghai, China). ROS inhibitor NAC was purchased from Topscience (Shanghai, China).

### Cell viability and apoptosis assays

Colon cancer cells were cultured in a 96-well microplate at a density of 5 × 10^3^ cells/well for 24 h. Cell viability was assessed with Cell Counting Kit-8 at indicated time post-treatment according to the manufacturer’s instructions. To estimate the viability of the cells, the absorbance of 450 nm (OD450) was measured with a 96-well plate reader. For analysis of apoptosis by Hoechst 33,258 (Invitrogen), colon cells were cultured on the coverslip of a chamber, rinsed with PBS, and then added in 500 μl of culture media containing 5 μg Hoechst 33,258, incubated at 37 °C with 5% CO_2_ for 15 min. Apoptosis was detected through microscopic visualization of condensed chromatin and micronucleation. For colony formation assays, equal number of cells were plated into 6-well plates after different treatments. 14 days after plating, colonies were visualized by crystal violet staining. Trypan blue (Beyotime, China) was also used for counting the number of dead cells. After collagenase digestion, centrifuging and suspending the cells with different treatments, trypan blue buffer was added into the cell suspension for 3 min. Then, the dead cells, stained with blue, were counted under light microscopy.

### ROS production assay

For intracellular reactive oxygen species (ROS) detection, cells were loaded with ROS specific fluorescent probe 2′,7′-dichlorofluorescin diacetate (DCFH-DA, Solarbio) and incubated for 30 min at 37 ℃. DCFH-DA can be oxidized into highly fluorescent dichlorofluorescein (DCF) in the presence of ROS, and fluorescence microscope was used to determine the intracellular ROS level. Rosup (Solarbio) was used as positive control.

### Western blotting

Protein samples were extracted with RIPA buffer (10 mM Tris–Cl (pH 8.0), 1 mM EDTA, 0.5 mM EGTA, 1% Triton X-100, 0.1% sodium deoxycholate, 0.1% SDS, 140 mM NaCl). The mitochondria isolation followed the instructions of the Cell Mitochondria Isolation Kit (Solarbio). Nuclear and cytoplasmic proteins were isolated using a Nuclear and Cytoplasmic Protein Extraction Kit (Solarbio). Equivalent protein samples (30 μg protein extract was loaded on each lane) were subjected to SDS-PAGE on 10% gel. The proteins were then transferred onto PVDF membranes (Millipore) and blocked with 5% non-fat milk for 1 h at room temperature. The membranes, probed with the indicated primary antibodies, were incubated at 4 °C overnight. Primary antibody was detected by binding horseradish peroxidase (HRP)-conjugated anti-rabbit or anti-mouse secondary antibody with an ECL plus kit. Detection was performed using the Odyssey infrared imaging system (LI-COR, Lincoln, NE). To detect AIF translocation, purified mitochondrial, cytoplasma and nucleus fractions were isolated, followed by Western blotting analysis.

### Real-time reverse transcriptase (RT) PCR

Total RNA was extracted with Tri-Reagent (TIANGEN BIOTECH, Beijing, China) according to the manufacturer’s protocol. The amount and purity of the RNA were determined by spectrophotometry, and 3 μg of RNA from the colon cancer cells after AKT inhibition were used in each RT reaction. The performance of realtime qPCR was performed by our previous report that we described on C1000 Thermal Cycler CFX96 Real-time PCR Detection System (Bio-Rad).

### Immunofluorescence staining

Cells were seeded in confocal dishes and treated as designated. After corresponding treatment, the cells were fixed in 4% paraformaldehyde for 30 min at room temperature, washed with phosphate buffered saline (PBS, pH 7.4) and then permeabilized with 0.4% TritonX-100 for 30 min. After blocking with 10% goat serum for 30 min, the cells were incubated with primary antibody overnight at 4 °C. Then the cells were washed with PBS and incubated with flurochrome conjugated secondary antibody against mouse or rabbit (proteintech, Wuhan, Hubei, China) for 1.5 h at room temperature. The specimens were then stained nuclei with DAPI for 5 min, and then observed by laser-scanning confocal microscope (Olympus, Tokyo, Japan).

### Flow cytometry

Human colon cancer cell lines HCT116 WT and HCT116 p53–/– were suspended in 1 × 10^5^ cells/ml, and 5 μl Annexin V and 5 μl propidium iodide staining solution were added to 100 μl of the cell suspension. Then 400 μl binding buffer was added to cell suspension again. After the cells were incubated at room temperature for 10 min in the dark, stained cells were assayed and quantified using a FACSort Flow Cytometer (Beckman Coulter, Brea, CA, USA). Cell debris was excluded from the analysis by an appropriate forward light scatter threshold setting. Compensation was used wherever necessary.

### Co‑immunoprecipitation

To detect the interaction among p53, SIRT6 and PARP1, about 6 μg of p53 antibodies was firstly added to 1 ml cell lysate. According to the manufacturer’s protocol, the mixtures were mixed on a rocker at ambient temperature for 2 h. The immunocomplexes were captured by the addition of protein A-agarose (Beyotime, Shanghai, China) mixed at 1:10 ratio, followed by incubation at ambient temperature for 1 h. The beads were washed 5 times and then collected by centrifugation at 12,000 rpm for 30 s. After the final wash, the beads were mixed with 20 μl of 2× loading buffer, heated at 95 °C for 10 min, and analyzed by Western blotting using p53 and PARP1 antibody.

### Xenograft mouse model and treatment

Female 5-week-old nude mice (Vital River, China) were housed in a sterile environment with micro isolator cages and allowed access to water and chow ad libitum. HCT116 WT cells were harvested, and 1 × 10^6^ cells in 0.2 ml of McCoy’s 5A modified medium were implanted subcutaneously into the back of athymic nude female mice. Mice were treated daily with SC66 at 25 mg/kg by i.p. injection every 3 days for 15 days, whereas the control mice were administered vehicle. Volume was calculated by the formula of 0.5 × length × width^2^. Mice were euthanized when tumors reached ~ 1.0 cm^3^ in size. Tumors were dissected and frozen in − 20 °C for further study. All mice were housed and maintained under specific pathogen-free (SPF) conditions. All animal studies were in accordance with institutional guidelines and approved by the Use Committee for Animal Care.

### Histopathology

For immunohistochemistry, tumors were fastened in 4% paraformaldehyde for 24 h. After dehydration, embedded in paraffin and sectioned by LEICA system according to standard protocols. Briefly, 1% hydrogen peroxide was used to blocked antigen retrieval with citric acid (pH 6.0) endogenous peroxidase activity. Primary antibody was applied and incubated with secondary antibodies conjugated to peroxidase-labelled dextran polymer. Sections not exposed to secondary antibody served as negative controls.

### Statistical analysis

Statistical analyses were performed using GraphPad Prism V software. All assays were repeated independently more than three times. Data are represented as mean ± SEM in the figures. *p* Values were calculated using the Student’s paired t-test.

## Results

### Targeting AKT suppressed colorectal cancer growth and induced apoptosis

**Fig. 1:**
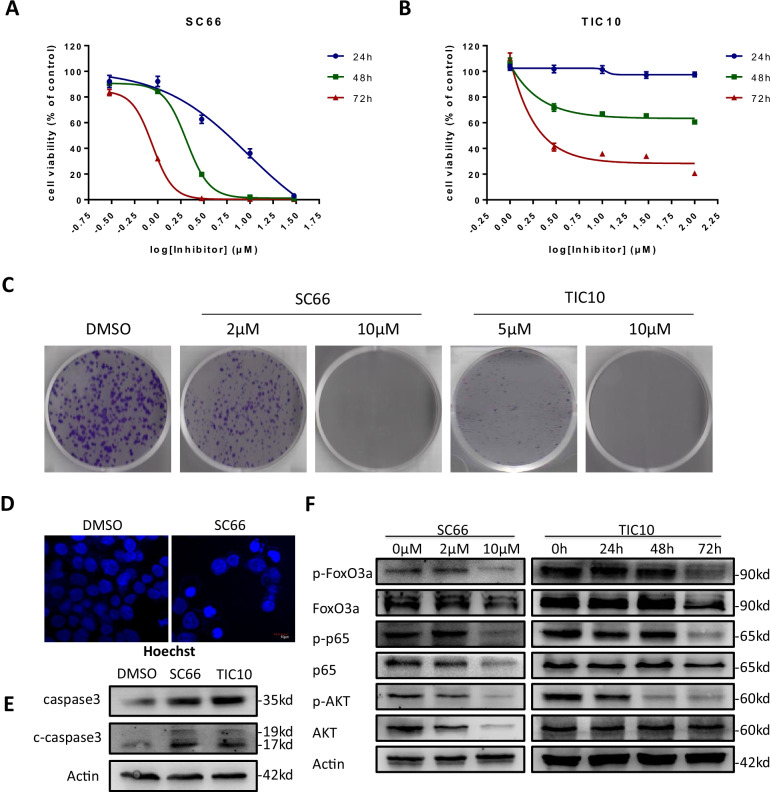
AKT inhibition promoted cell death in colorectal cancer cells. **a**, **b** Cell viability of HCT116 was analyzed by CCK-8 assay after administration of SC66 (0.33 μM, 1 μM, 3.3 μM, 10 μM and 33 μM) or TIC10 (1 μM, 3.3 μM, 10 μM, 33 μM and 100 μM) at the time points of 24 h, 48 h and 72 h. **c** Colony formation assay by crystal violet staining in HCT116 treated with SC66 (2 μM, 10 μM) and TIC10 (5 μM, 10 μM) after 14 days. **d** Hoechst 33,258 staining was performed after 10 μM of SC66 treatment for 24 h **e** After HCT116 cells were treated with 10 μM of SC66 and TIC10 for 24 h and 48 h respectively, the level of total-caspase3 and cleaved-caspase3 was tested via western blotting. **f** The expression levels of p-Akt (S473), p-FoxO3a (S253) and p-p65(S276) were detected after the treatment of SC66 (2 μM, 10 μM) for 24 h and TIC10 (10 μM) for 24 h, 48 h or 72 h in HCT116

To study how AKT inhibitors SC66 and TIC10 affecting CRC proliferation, cell viability was detected by CCK-8 assay under a gradient in concentration of SC66 and TIC10 treatment for 24, 48 and 72 h. Cell viability decreased remarkably, while SC66 exhibited a dose dependent manner (Fig. 1a) and TIC10 was time dependent (Fig. 1b). Colony forming assay showed similar results that SC66 and TIC10 significantly suppressed colony formation (Fig. 1c). Hoechst33258 staining was performed and chromatin condensation was observed in HCT116 cells after treated with SC66 (Fig. 1d) and TIC10 (Additional file 1: Fig. S1a). The expression of total-caspase 3 and cleaved-caspase 3 was detected increased in HCT116 after application of SC66 and TIC10 (Fig. 1e). Above results confirmed apoptotic cell death was taking place in HCT116 cells. Meantime, AKT and its downstream molecules FoxO3a and p65 might be dephosphorylated after AKT inhibition (Fig. 1f). Interestingly, SC66 inhibits AKT activity by down-regulating AKT protein level, which is probably due to SC66 functioned through facilitating Akt ubiquitination [38].

### AKT inhibition triggers not only apoptosis but also parthanatos and pro-survival autophagy

**Fig. 2:**
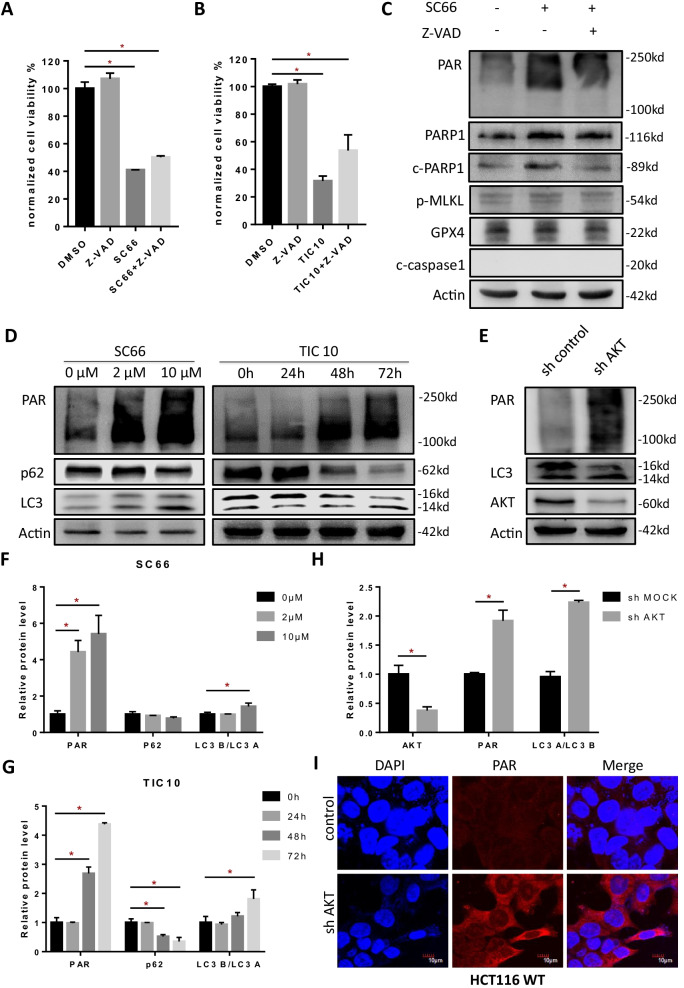
AKT inhibition triggers parthanatos and autophagy. **a**, **b** Cell viability of HCT116 was detected by CCK-8 assay after administrated 2 μM SC66 (**a**) and 10 μM TIC10 (**b**) for 48 h with or without 10 μM Z-VAD (caspase inhibitor). **c** The expressions of cleaved-caspase1, GPX4, p-MLKL (S358), PARP1, cleaved PARP1 and PAR in HCT116 were tested after administrated 10 μM SC66 with or without Z-VAD. **d** Western blotting assay tested the level of LC3, p62 and PAR in HCT116 treated with SC66 or TIC10. **e** Western blotting assay tested the level of AKT, LC3 and PAR in HCT116 after knock down of AKT. **f**, **g**, **h** Qualitative analysis of LC3, p62 and PAR levels under SC66 (**f**), TIC10 (**g**) and AKT knock down (**h**). **i** Immunofluorescent analysis of the PAR polymer (red) accumulation in HCT116 with knock down of AKT

**Fig. 3:**
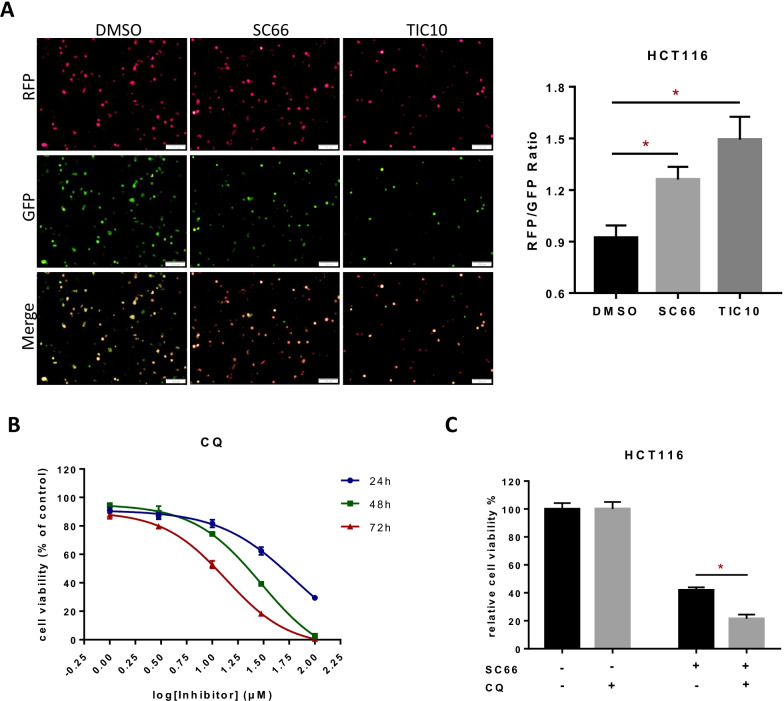
AKT inhibition triggered autophagy is not a cell death pathway but a pro-survival adaption. **a** The ratio of RFP/GFP fluorescent was analyzed after treated with 10 μM of SC66 and TIC10 for 24 h and 48 h respectively. **b** Cell viability of HCT116 was analyzed by CCK-8 assay after administration of CQ (1 μM, 3.3 μM, 10 μM, 33 μM and 100 μM) at the time points of 24 h, 48 h and 72 h.** c** Cell viability of HCT116 was detected by CCK-8 assay after administrated 2 μM SC66 for 48 h with or without 10 μM CQ. *p < 0.05

In consideration whether AKT promotes cell survival only via apoptotic cell death, we then assessed a combination of AKT inhibitor and caspase inhibitor (Z-VAD-FMK) which is widely used in researches to block apoptosis [39]. As a result, dead cells ratio reduced after administration of Z-VAD-FMK (Additional file 2: Fig. S2a, b) but cell viability only showed a partial recovery (Fig. 2a, b), which implied that AKT inhibition also play a role in caspase-independent non-apoptotic cell death. Consequently, after treating HCT116 cells with SC66 and Z-VAD-FMK we detected the markers of non-apoptotic cell death including cleaved-caspase1 [40], GPX4 [41], p-MLKL [42] and PAR polymer, the classic markers of pyroptosis, ferroptosis, necroptosis and parthanatos respectively and the results showed that parthanatos was positive while pyroptosis, ferroptosis and necroptosis were negative (Fig. 2c). So far, after AKT blocked by small molecule inhibitors or genetic knock down in HCT116, we discovered PAR polymer accumulated and LC3-II/LC3-I ratio increased (Fig. 2d, e) via western blotting assay, and corresponding qualitative analysis were showed below (Fig. 2f–h) which indicated that AKT inhibition may induce parthanatos and autophagy. Consistently, similar result (Additional file 2: Fig. S2d) that PAR polymer accumulation was also observed in another colon cancer cell line, RKO, which express wild type p53 protein as HCT116 does. Immunofluorescence results confirmed that knock down of AKT induced PAR polymer synthesized (Fig. 2i). RFP-GFP-LC3 plasmid was transfected into HCT116 cells, after the treatment of AKT inhibitors, GFP degradation and RFP/GFP elevation were observed, which implied the activation of autophagy (Fig. 3a). GFP-LC3 was also transfected and punctate aggregated was observed after administration of SC66 and TIC10 (Additional file 2: Fig. S2e). Chloroquine (CQ) is an effective autophagy inhibitor and according to the growth inhibitory curve we tested (Fig. 3b), 24 h of CQ (10 μM) treatment was selected for the following assay. However, a combination of SC66 and CQ has failed to rescue cell viability (Fig. 3c), and it revealed that AKT inhibition induced autophagy is not a cell death pathway but a pro-survival adaption. Interestingly, autophagy inhibitors dramatically reduced AKT inhibition induced PAR polymer accumulation (Additional file 2: Fig. S2f, g), the reason of PAR polymer decrease might be the inhibition of autophagy, which enhances apoptosis [43] and activates caspase kinase thus repressing parthanatos by cleaving PARP1 [44].

### Blocking PARylation activity abrogated parthanatos induced by AKT inactivation.

**Fig. 4:**
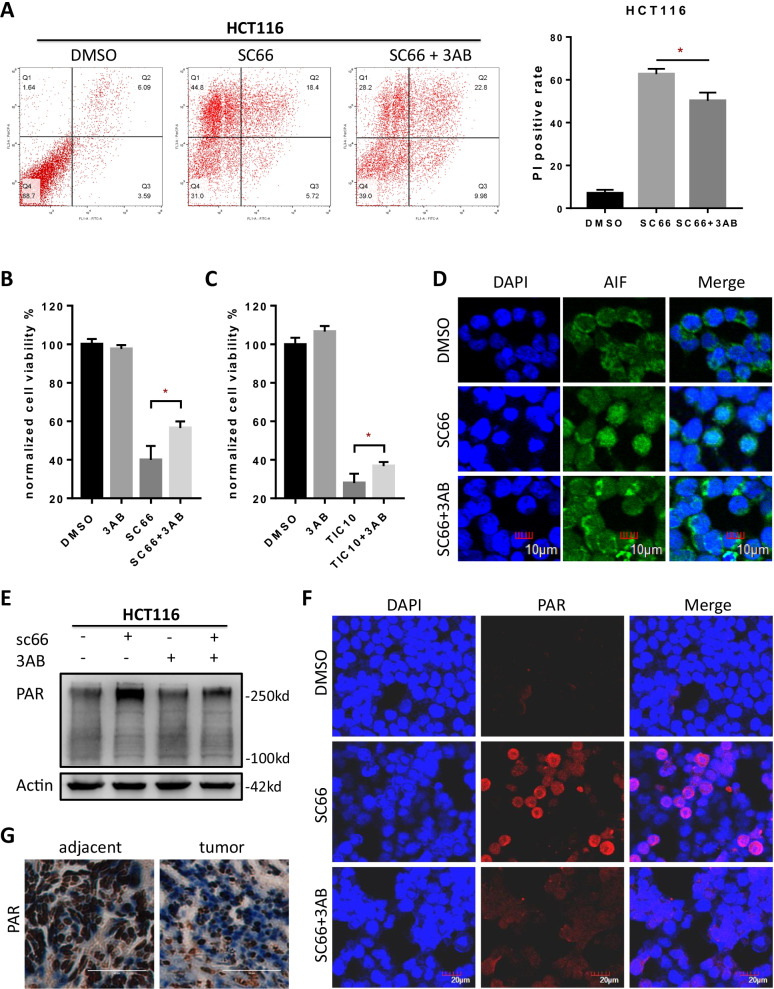
Blocking of PARP1 activity abrogated AKT related parthanatos. **a** Annexin V/PI staining and flow cytometry analysis of cell death under 10 μM SC66 treatment for 24 h with or without PARP1 inhibitor, 3AB (10 μM). **b**, **c** Cell viability of HCT116 was detected by CCK-8 assay after administrated 2 μM SC66 (**b**) and 10 μM TIC10 (**c**) for 48 h with or without 10 μM 3AB. **d** Immunofluorescent analysis of AIF (green) translocation under SC66 (10 μM) treatment with or without 3AB (10 μM). **e**, **f** The expressions of PAR polymer in HCT116 were tested after administrated SC66 with or without 3AB by western blotting assay (**e**) and immunofluorescent stain (**f**) of the PAR polymer (red). **g** Immunohistochemistry analysis shows PAR polymer expression in human CRC tissue and adjacent tissue. *p < 0.05

To investigate the role of PARP1, which conducts more than 90% of the total PARylation activity in the induction of parthanatos, in AKT inhibition induced parthanatos, loss function study by blocking PARP1. Flow cytometry results showed that SC66 induced PI positive necrotic cell counts could be reduced by PARP1 inhibitor, 3AB (Fig. 4a). Since parthanatos was regarded as a form of regulated necrosis and positive PI stain was regarded as its feature [7]. Phosphatidylserine externalization on outer plasma membrane is a classic sign of necrotic cell death [45]. So the principle of it might be due to the phosphatidylserine externalization caused by PAR polymer. CCK-8 assay and Trypan blue staining showed that administration of 3AB, partly rescued cell viability (Fig. 4b, c) and decreased dead cells rate (Additional file 2: Fig. S2a, c) induced by AKT inhibitor. To confirm whether the phenotypes of AKT inhibition induced parthanatos were mediated by PARP1 activity. Firstly, translocation of AIF into the nucleus induced by SC66 was observed under confocal microscopy in HCT116 cells and which can be reversed by applicating 3AB (Fig. 4d, Additional file 1: S1e). Consistently, via western blotting assay (Fig. 4e) and immunofluorescence staining (Fig. 4f), PARP1 inhibition dramatically blocked AKT related PAR polymer accumulation. Besides, immunohistochemistry stain showed the level of PAR polymer was significantly reduced in tumor tissue compared with adjacent tissue (Fig. 4g), indicating that inducing parthanatos could be a potential strategy for clinical treatment. Taken together, above results demonstrated that AKT related parthanatos in HCT116 was PARP1 dependent.

### P53 is indispensable in AKT inhibition induced parthanatos

**Fig. 5:**
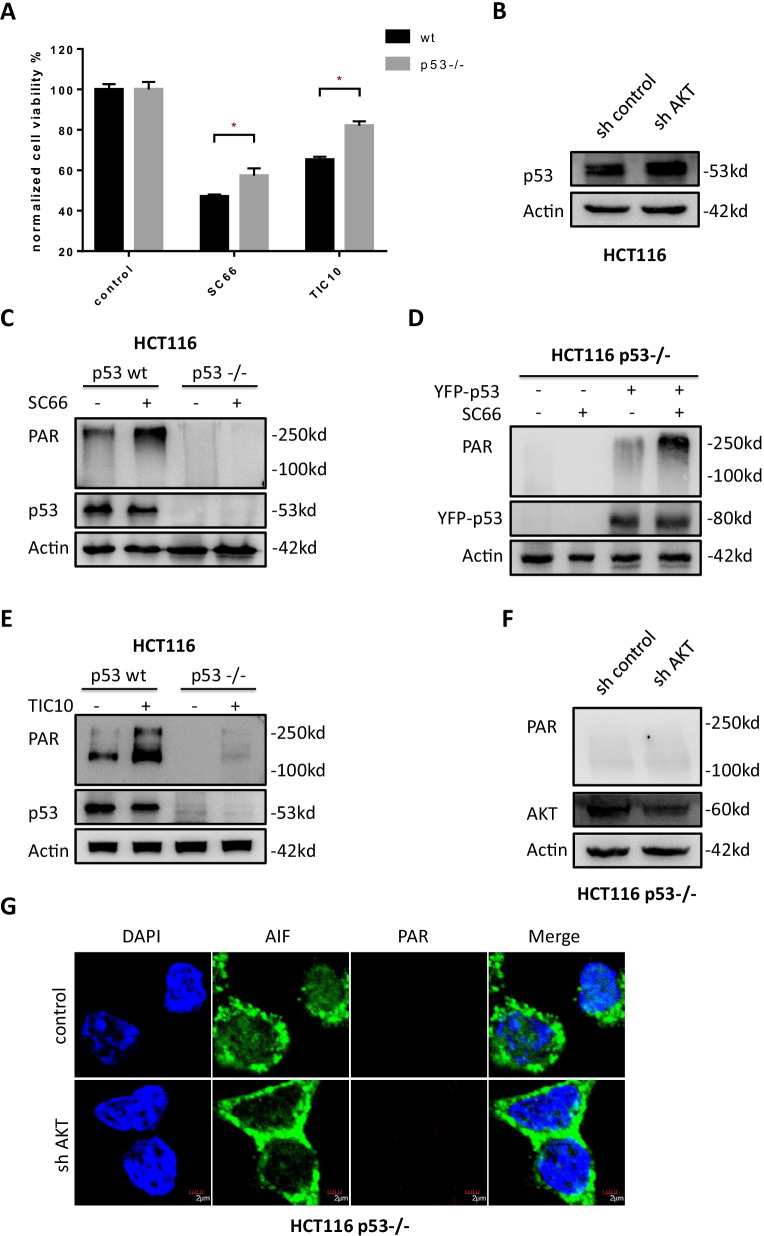
AKT inhibition induced p53 mediated parthanatos. **a** Cell viability of HCT116 wild type and p53–/– cells was detected by CCK-8 assay after administrated 2 μM SC66 and 10 μM TIC10 for 48 h. **b** Western blotting assay tested the level of p53 in AKT knockdown cells. **c**,** d** After treated with 10 μM SC66 for 24 h, the cellular PAR polymer level in HCT116 wild type and p53–/– cells (**c**) and in HCT116 p53–/– with or without transfection of YFP-p53 (**d**) were tested by Western blotting. **e** PAR polymer level in HCT116 wild type and p53-/- cells was tested after treated with 10 μM TIC10 for 48 h. **f**, **g** Western blotting assay (**f**) and immunofluorescent analysis (**g**) of the PAR polymer accumulation after knockdown of AKT. *p < 0.05

**Fig. 6:**
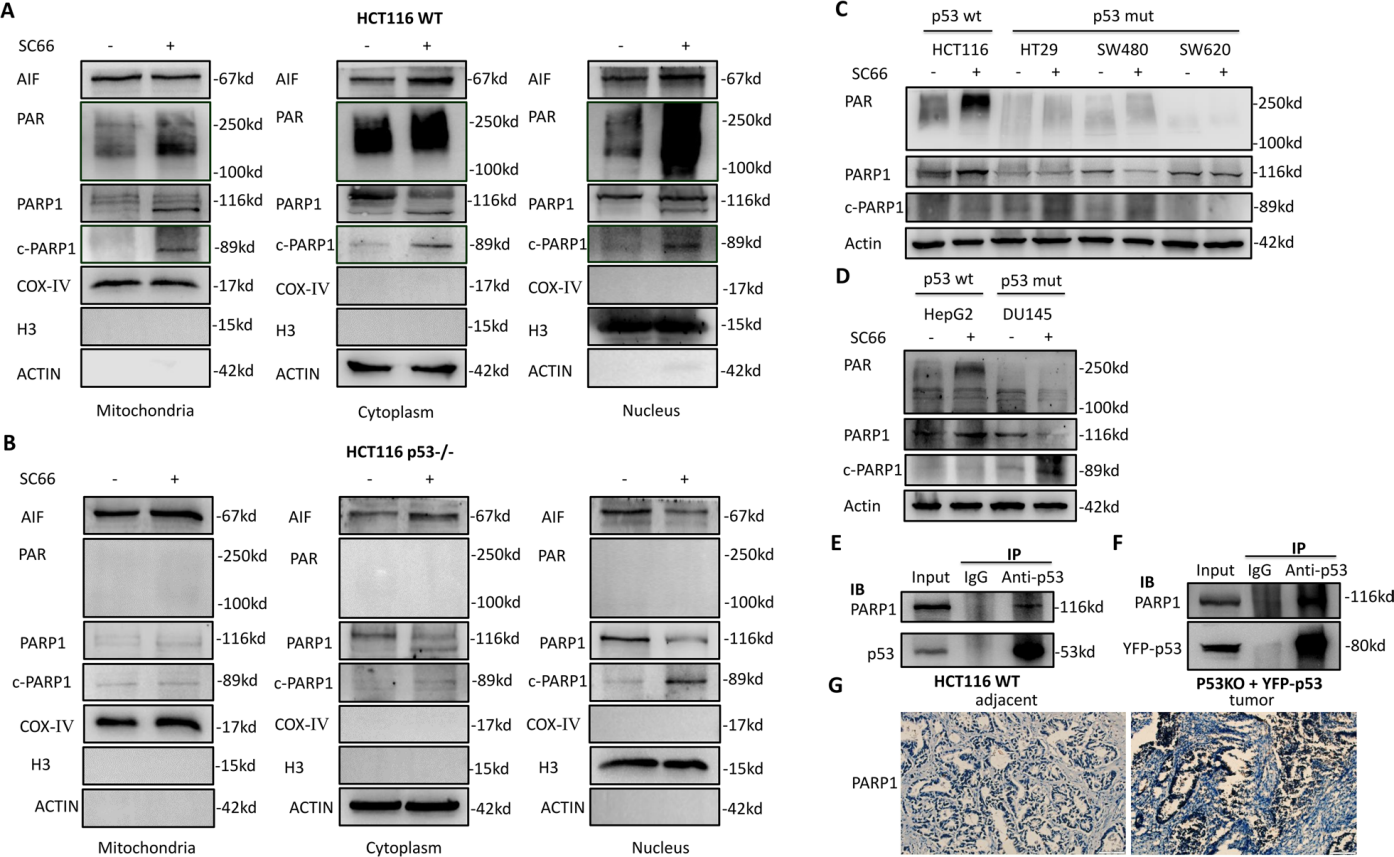
p53 activates PARP1 in AKT inhibition triggered parthanatos. **a**, **b** After SC66 treatment for 4 h, mitochondrial, cytoplasmic and nuclear fractions were isolated from HCT116 wild type (**a**) or HCT116 p53–/– cells (**b**) and subjected to western blot for detection of the protein levels of PAR, cleaved PARP1, PARP1 and AIF. COX IV, β-actin and H3 was used as the loading control of mitochondria, cytoplasm and nucleus respectively. **c** Western blotting assay tested the PAR polymer level in different colorectal cancer cells with or without p53 mutation. **d** Western blotting assay tested the PAR polymer level in HepG2 cells (wild type p53) and DU145 (p53 mutated).** e** Endogenous co-immunoprecipitation was performed in HCT116 cells. **f** Exogenous co-immunoprecipitation was performed in p53 null cells transfedted with YFP-p53. **g** A comparison of PARP1 expression level between CRC tissues and adjacent normal tissues

Then we assessed the role of the p53 in the process of AKT inhibition induced parthanatos, CCK-8 assay showed that both HCT116 WT and HCT116 p53–/– cells were observed decrease in cell viability but HCT116 p53–/– cells had a higher survival rate than HCT116 WT cells (Fig. 5a). After treated with AKT inhibitor, p53 did not increase on mRNA level (Additional file 3: Fig. S3a) or protein level (Fig. 5c), but p53 level increased in AKT knockdown cells (Fig. 5b). Compared with p53 wild type HCT116 cells, PAR polymer accumulation was not able to be induced by SC66 in HCT116 p53–/– cells (Fig. 5c) while, after transfected with YFP-p53 overexpression plasmid PAR polymer accumulation can again be observed under administration of SC66 (Fig. 5d). Similar result was observed after treating HCT116 WT and p53–/– cells with TIC10 (Fig. 5e). Consistently, unlike in HCT116 WT cells, after knock down of AKT in HCT116 p53–/– cells (Fig. 5f), PAR polymer accumulation and AIF translocation into the nucleus were not able to be observed (Fig. 5g). In addition, AIF protein distribution in mitochondria, cytoplasm and nuclear was detected by western blot. After SC66 treatment in HCT116 WT cells, AIF protein level in mitochondrial fraction was decreased, and with predominant increase in nucleus fraction (Fig. 6a), on the contrary, AIF protein in nucleus fraction was not increased in HCT116 p53–/– cells (Fig. 6b). According to a online database cBioportal (https://cbioportal.org/) [46], p53 mutation occurs in colon cancer with a high frequency at about 73% (Additional file 4: Fig. S4a), especially in R273 site (Additional file 4: Fig. S4b). Interestingly, all HT29, SW480 and SW620 harbour p53 R273H mutation, and PAR polymer accumulation can not be induced in these cells with p53 loss-of-function mutation (Fig. 6c). Similarly, under the administration of SC66, PAR polymer accumulation was happened in HepG2, a hepatocellular carcinoma cell line with wild type p53, but not occurred in DU145, a prostatic cancer cell line with mutated p53 (Fig. 6d). In summary, AKT related parthanatos is p53 dependent and if in absence of p53 normal function parthanatos was failed to be triggered by AKT inhibition, which may partly explained why it is harder to repress CRC in the patients with p53 mutation.

### P53 activates PARP1 directly to promote parthanatos while mutant p53 losses this function

In consideration that PARP1 activity was enhanced by p53, we then assessed the relationship between p53 and PARP1. In the western blotting image, multiple isoforms of PARP1 were seen, the 116 kd bands represent the full length PARP1, which indicates PARP1 activity, and the smaller 89 kd bands represent the cleaved-PARP1, which indicates the occurrence of apoptosis [47, 48]. Although a part of PARP1 was cleaved after SC66 treatment, protein level of nucleus PARP1 was upregulated in HCT116 WT cells (Fig. 6a). On the contrary, nucleus PARP1 in HCT116 p53–/– cells was not and the reason of full length PARP1 decrease is probably due to PARP1 cleavage (Fig. 6b). Moreover, protein levels of PAR also confirmed that the activation of PARP1 was closely associated with p53 (Fig. 6a, b). Consistently, AKT inhibition triggered PARP1 activation was only observed in HCT116 cells with wild type p53 but not in p53 mutant cells like HT29, SW480 and SW620 (Fig. 6c). Similar results were also observed beyond CRC cell lines, PARP1 was activated by SC66 treatment in HepG2 cells with wild type p53, but not in DU145 cells, with mutant p53 (Fig. 6d). Next, in order to investigate whether p53 binds to PARP1 directly, co-immunoprecipitation assay was performed in HCT116 WT cells and HCT116 p53 null cells transfected with YFP-p53 overexpression plasmid and the results showed that both endogenous and exogenous interaction between p53 and PARP1 were detected (Fig. 6e, f). Exogenous co-immunoprecipitation was also performed in HEK293T (Additional file 3: Fig. S3b) and the reciprocal experiment with anti PARP1 antibody and IB with anti-p53 antibody was performed in HCT116 cells (Additional file 3: Fig. S3c). Besides, PARP1 protein level is significantly up-regulated in CRC cancer tissues compared with normal tissues, according to immunohistochemistry results (Fig. 6g) and a on line database UALCAN (ualcan.path.uab.edu/index.html) [49], which indicating PARP1 is also a potential therapeutic target to inducing parthanatos in CRC (Additional file 4: Fig. S4c). In general, inhibition of AKT triggers parthanatos by regulating p53 and then activating PARP1.

### ROS promotes AKT inhibition induced parthanatos

**Fig. 7:**
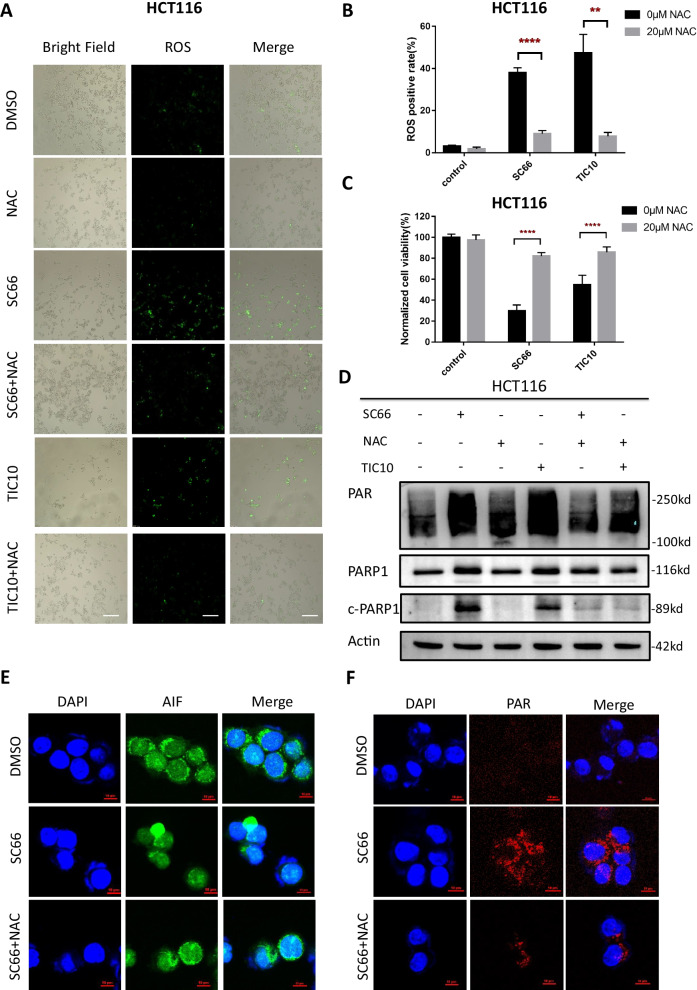
ROS promotes AKT inhibition induced parthanatos. **a** ROS production in HCT116 was tested by fluorescence microscope after different treatments for 12hm. **b** ROS positive cells rates after treatment. **c** Cell viability of HCT116 was detected by CCK-8 assay after administrated 10 μM SC66 and 10 μM TIC10 for 24 h with or without 20 μM NAC. **d** The expressions of PAR polymer and PARP1 in HCT116 were tested after administrated 10 μM SC66 and 10 μM TIC10 for 24 h with or without 20 μM NAC by western blotting assay. **e** Immunofluorescent analysis of AIF (green) translocation under SC66 (10 μM) treatment with or without NAC (10 μM). **f** Immunofluorescent stain for the PAR polymer (red). **p < 0.01, ****p < 0.0001

ROS accumulation is closely related to various types of cell death [50]. After treated with AKT inhibitor (Additional file 1: Fig. S1b) or AKT knock down (Additional file 1: Fig. S1c), ROS production was observed in HCT116 cells. To further explore the effects of ROS accumulation during the AKT inhibition-induced parthanatos, NAC (N-acetylcysteine), a classical ROS inhibitor [51], was used for the following experiments of ROS, with a dose of 20 μM in vitro [52]. ROS production assay showed that after inhibiting AKT by SC66 or TIC10, ROS produced more, which could be reduced by co-treatment of AKT inhibitor and NAC (Fig. 7a, b). Besides, cell death induced by targeting AKT could be rescued by inhibiting ROS (Fig. 7c) and after inhibiting both ROS and AKT, expression level of PARP1 decreased significantly compared with that of inhibiting AKT only (Fig. 7d). Additionally, confocal microscopy was utilized to observe the translocation of AIF into the nucleus (Fig. 7e). It is shown that translocation of AIF into the nucleus induced by SC66 could be reversed by co-treatment with NAC. ROS inhibition also dramatically blocked AKT related PAR polymer accumulation, which was verified via western blotting assay (Fig. 7d) and immunofluorescence staining (Fig. 7f). Taken together, ROS could promote parthanatos induced by AKT inhibition, and targeting and elevating ROS produced by inhibiting AKT might be served a novel insight for the anti-tumor therapeutics.

### SIRT6 promotes AKT inhibition induced parthanatos

**Fig. 8:**
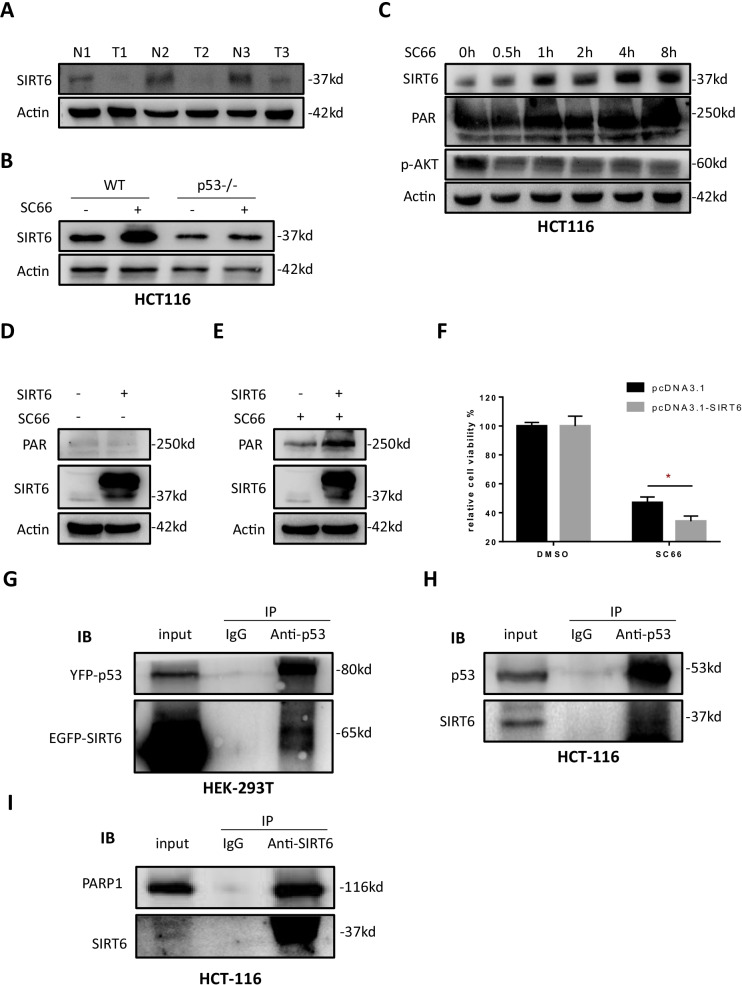
SIRT6 promotes AKT inhibition induced parthanatos. **a** SIRT6 expression in human CRC tumor tissue (T) and adjacent normal tissue (N). **b** SC66 (10 μM) treatment for 24 h elevated SIRT6 expression in HCT116 cells. **c** SC66 (10 μM) treatment at the time points of 0 h, 0.5 h, 1 h, 2 h, 4 h and 8 h elevated the expression of SIRT6 and PAR polymer in a time dependent manner. **d**, **e** Western blotting assay tested the levels of PAR polymer and SIRT6 without (**d**) or with (**e**) SC66 (10 μM) treatment after SIRT6 overexpression. Empty plasmid (pcDNA 3.1) was used as control. **f** CCK-8 tested the SIRT6 overexpressed cells viability after treated with 2 μM SC66 for 48 h. **g** Exogenous co-immunoprecipitation was performed in HEK293T cells transfedted with YFP-p53 and EGFP-SIRT6. **h** Endogenous co-immunoprecipitation of SIRT6 and p53 was performed in HCT116 cells. **i** Endogenous co-immunoprecipitation of SIRT6 and PARP1 was performed in HCT116 cells

SIRT6 was observed decreased in colorectal cancer tissue compared with tumor adjacent (Fig. 8a) and a similar result was also showed by the online database (Additional file 4: Fig. S4d). SIRT6 expression elevated significantly after AKT inhibition, especially in HCT116 cells (Fig. 8b), and which showed a similar trend to PAR polymer (Fig. 8c). Overexpression of SIRT6 did not induce parthanatos directly (Fig. 8d) but enhanced AKT inhibition induced PAR polymer formation (Fig. 8e) which dramatically reduced cell viability (Fig. 8f). Moreover, co-immunoprecipitation assay was performed and the results showed that both exogenous and endogenous interaction between SIRT6 and p53 was observed (Fig. 8g, h). And to explore whether SIRT6 interacts with PARP1, co-immunoprecipitation assay was performed in HCT116 WT cells (Fig. 8i), which showed the endogenous interaction between SIRT6 and PARP1. Together, we speculate that SIRT6 promotes AKT inhibition induced p53 dependent parthanatos, by forming a SIRT6/p53/PARP1 complex.

### AKT inhibition exhibited a p53 dependent antitumor effect via parthanatos in vivo

**Fig. 9:**
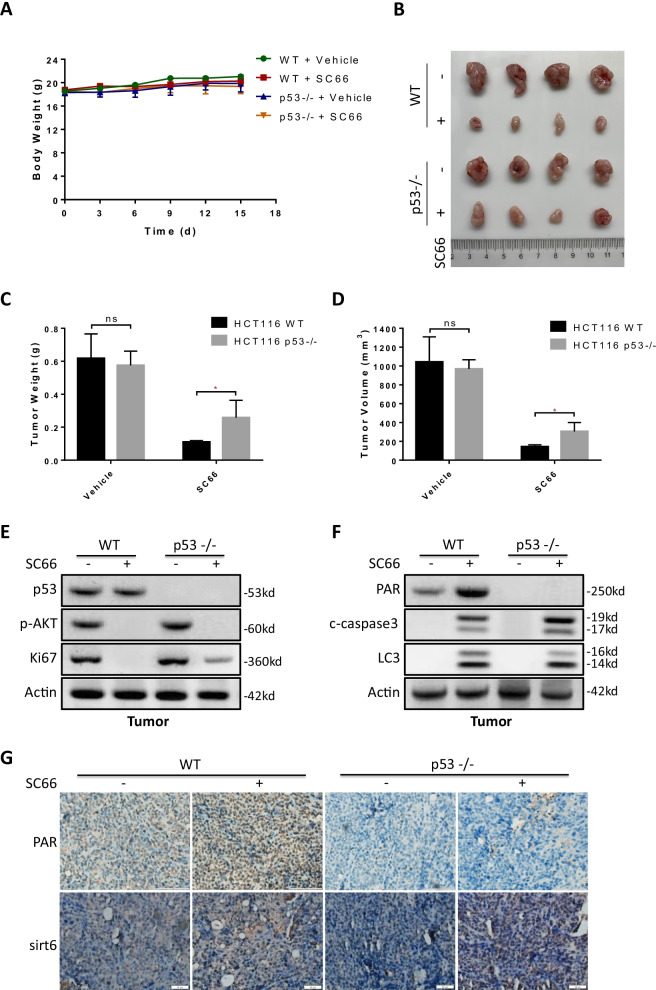
The antitumor effect of AKT inhibition depends on p53 in vivo. Nude mice were injected s.c. with 1 × 10^6^ HCT116 wt or p53–/–, and mice were treated with SC66 at 25 mg/kg by i.p. injection every 3 days for 15 days. **a** The body weight of mice in each group during treatment. **b**, **c**, **d** Representative tumors at the end of the experiment (**b**) Tumor weight (**c**) and tumor volume (**d**) were measured after treated with SC66. **e**, **f** Western blotting assay tested the levels of p53, p-AKT, ki67 (**e**), PAR polymer, cleaved-caspase3 and LC3 (**f**) in the harvested tumor. **g** Immunohistochemistry analysis the levels of PAR polymer and SIRT6 in the tumors. *p < 0.05

Next, we continue investigating whether AKT inhibition triggered p53 mediated parthanatos is necessary for antitumor activity in xenograft models. We established xenograft mice models by subcutaneous injection of HCT116 WT and HCT116 p53–/– cells. After treatment, there was no significant change in the body weight of the mice (Fig. 9a). Tumor growth was evidently inhibited in both wild type and p53–/– HCT116 cells after SC66 treatment. However, in HCT116 p53–/– groups, the inhibition rate of tumor weight and volume were significantly lower than that in HCT116 WT groups (Fig. 9b–d). The levels of p53, p-AKT, ki67, PAR polymer, cleaved-caspase3 and LC3 in the harvested tumor were tested by western blotting (Fig. 9e, f) and the level of PAR polymer and SIRT6 were also tested by immunohistochemistry (Fig. 9g). It indicates that AKT inhibition mediates not only p53 independent apoptosis but also p53 dependent parthanatos in vivo. The p53 dependent parthanatos importantly contributed to AKT inhibition induced cell death, which should be regarded as a potential therapeutic strategy for future clinical treatments.

## Discussion

Colorectal cancer is one of the most common causes of cancer death with the incidence rate of 38.7 per 100,000 persons and the mortality rate of 13.9 per 100,000 persons [53]. The survival rate of CRC patients is closely related to the stage of the cancer, sadly, approximately 35% of CRC patients were found with metastasis when diagnosed which seriously influenced the prognosis [54]. Cytotoxic chemotherapy agents like 5-Fluorouracil, oxaliplatin and other drugs, which tends to kill rapidly proliferating cells non-selectively, are wildly used in clinical treatments for advanced CRC. However, due to the lack of specificity and the emergence of drug resistance the utility of these agents may be limited [55]. Besides, CRC frequently carries various oncogenes and tumor suppressor genes mutation including but not limited to TP53, KRAS, BRAF and PI3KCA, which makes the circumstances more complicated [56–59]. Therefore, targeted therapies were developed rapidly, and had been applied in combination with first line chemotherapy. Targeted therapy drugs like vascular endothelial growth factor (VEGFA) inhibitor bevacizumab, epidermal growth factor receptor (EGFR) inhibitor cetuximab and panitumumab have been granted by Food and Drug Administration (FDA) for metastasis CRC treatment [60, 61]. Interestingly, PI3K-AKT pathway was identified as a pro-survival factor contributing to drug resistance and it shows close relationships to all common mutations in CRC. Mechanically, PI3K-AKT pathway can be activated by both VEGFA or EGFR hyperactivation and PI3KCA mutation, and it can also regulate downstream molecules like RAF and p53 [55, 62]. Therefore, activation of AKT pathway is very likely to play a critical role in metastatic CRC and targeting AKT may be a potential therapeutic strategy.

Previous studies have suggested that targeting AKT showed significant preclinical anti-tumor effects in CRC and many other malignant tumors like pancreatic cancer [63, 64]. AKT inhibitor has also been proved to successfully sensitize 5-Fu resistance CRC cells to chemotherapy [65]. However, previous researches were mainly focused on AKT related apoptosis, while AKT associate non-apoptotic cell death has not been well studied. Although, a few studies mentioned that interfering PI3K/AKT signaling pathway may related with pyroptosis, ferroptosis and necroptosis in non-tumor diseases like hepatic ischemia reperfusion [66], hypoxic-ischemic brain damage [67] and in cardiac myofibroblasts [68] respectively, seldom research links AKT inhibition to above non-apoptotic cell death in neoplastic disease.

In the present study, we investigated whether AKT pathway takes part in non-apoptotic cell death in human CRC and the occurrence of parthanatos was observed. Specific molecules markers of pyroptosis, ferroptosis, necroptosis and parthanatos were tested and results showed that pyroptosis, ferroptosis and necroptosis were tested negative, while, the markers of PARP1 activation and parthanatos initiation, the accumulation of PAR polymer, were tested positive. During parthanatos, AIF translocation from mitochondria to nucleus, another sign of parthanatos [10], caused by massive DNA damage induced PARP1 hyperactivation and excessive synthesis of PAR polymer, was also been observed in our study. Besides, we also observed a pro-survival autophagy happened under the inhibition of AKT. Mechanically, we identified p53 as a critical downstream of AKT kinase in parthanatos, which links AKT inhibition with PARP1 hyperactivation. As we know, AKT kinase suppresses p53 protein by over phosphorylate MDM2, a classic antagonists of p53, which promotes p53 degradation [23]. Parthanatos is a form of regulated necrosis requires PARP1 activation in the process [6]. Although no direct data at the molecular level was proved, study revealed PARP activity is evidently dependent on p53 [69]. Consistently, our data showed that p53 protein level was obviously increased in AKT knockdown cells and after AKT inhibition, even though PARP1 was observed partly cleaved by caspases, a sign of apoptosis [70], but the level of remaining full length PARP1 was still significant higher than that in control cells, indicated both p53 and PARP1 were elevated and activated by blocking AKT. Interestingly, PARP1 was failed to be activated in p53 knock out and p53 mutant CRC cells, which indicated PARP1 activation and parthanatos require the function of wild type p53. Direct interaction between p53 and PARP1 was also confirmed by co-IP assay in our study. Comparing with other studies, we put forward that AKT inhibition induces parthanatos for the first time, and partly illuminated the underlying mechanism. Moreover, mutations in p53 are frequently found in human CRC, missense mutation like R273H and R175H promote submucosal invasion [71]. Mutation and absence of wild-type p53 were reported to accelerate the late stage of colorectal cancer progression through various oncogenic pathways [72]. Our findings suggested that lacking of parthanatos activity, caused by p53 mutation or deletion, might be another mechanism of poor prognosis in patients without normal p53 function. Thus, in further studies, finding a way to trigger parthanatos in patients with p53 mutation may play an important role in tumor suppression.

**Fig. 10:**
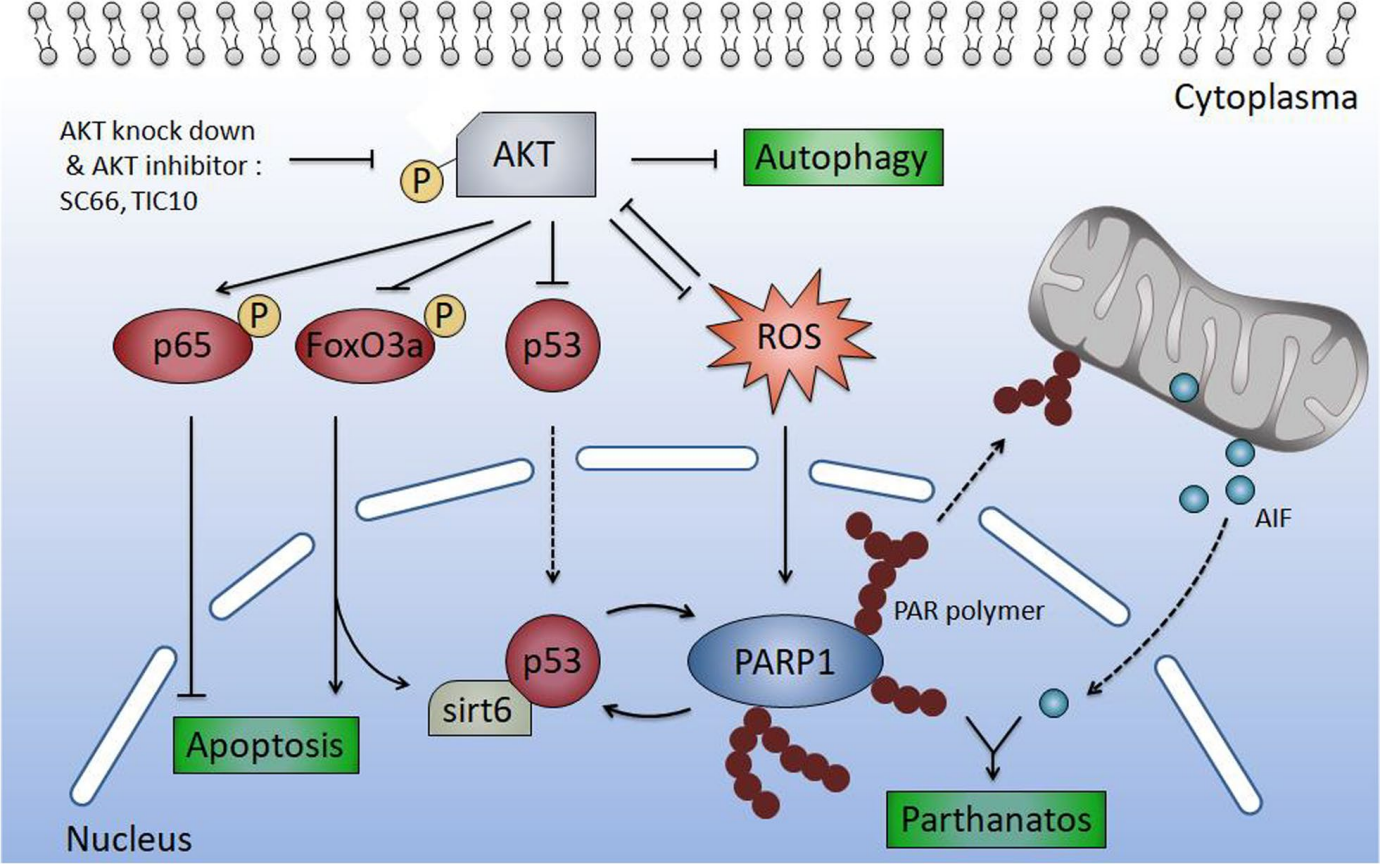
Hypothetical molecular mechanism of AKT inhibition induced parthanatos

The molecular mechanism and biological process of parthanatos present great complexity. PARP1 hyperactivation mediated parthanatos is usually triggered by oxidative injury like reactive oxygen species (ROS) accumulation [16]. It is reported that pharmacologically blocking AKT pathway promotes ROS production [73], and in our study, similarly, cellular ROS was observed increased significantly by treating with AKT inhibitor and pathanatos induced by targeting AKT could be increased by ROS (Fig. 7a–f). In addition, after inhibition of AKT, dephosphorylation of the downstream molecules FoxO3a and p65 were observed in the present study, which indicated the activation of FoxO3a [35] and inactivation of p65-NFκB [74] and it is also reported that both activated FoxO3a and inhibition of p65 can induce ROS generation [75, 76]. Interestingly, ROS was also reported to promote AKT ubiquitination and degradation [77], thus we speculate the existence of a potential feedback loop between AKT and ROS, which constantly promotes ROS production and AKT degradation. Moreover, it has been reported that PARP1 has several effects on p53, including the stabilization and poly(ADP-ribosyl)ation of p53 which modulates its DNA binding properties, transcriptional function, replication-associated recombination and specific protein–protein interactions, besides that, activated p53 was also reported to induce the function of PARP1 but its mechanism remains unknow [78, 79]. In present study, we observed that PARP1 activation is p53 dependent, and which is very likely to be realized by direct interaction. Thus, we speculated that the occurrence of p53-PARP1 positive feedback loop may also happen after AKT inhibition. Meanwhile, in this study, loss of SIRT6 expression was observed in tumor tissue compared with adjacent tissue and we found SIRT6 expression elevated significantly after inhibition of AKT. Overexpression of SIRT6 promoted AKT inhibition induced cell death and PAR polymer synthesis. Direct interaction between SIRT6 and p53 was also proved. So we hypothesize that SIRT6 also took part in AKT inhibition induced p53 depended parthanatos. Together, hyperactivation of AKT kinase, p53 loss of function and upregulation of PARP1 were observed in many types of neoplasms including CRC. Therefore, like in our research, finding a way to suppress aberrant AKT kinase, stabilize p53 protein and activate PARP1 was a promising strategy to overcome apoptosis resistance and induce non-apoptotic parthanatos in cancer treatment. After AKT inhibition, p53-SIRT6-PARP complex formation and ROS production may amplify the cell death signal and trigger the process of parthanatos (Fig. 10).

In conclusion, for the first time, our results demonstrated that inhibition of AKT triggers not only apoptosis but also a p53/SIRT6/PARP1 dependent parthanatos, which may uncover novel therapeutic strategies for CRC treatment.

## Conclusions

Targeting AKT suppresses tumor growth through inducing apoptosis, which has been wildly explored and served as therapeutic targets for cancer. However, whether other forms of cell death occurring during inhibiting AKT is poorly understood. In the present study, we identified that inhibiting AKT could trigger parthanatos thus leading to colon cancer growth suppression. We also found that after pharmacological or genetic AKT inhibition, p53 interacted with SIRT6 and PARP1 directly to activate it, and promoted the formation of PAR polymer. Subsequently, PAR polymer transported to outer membrane of mitochondria and resulted in AIF releasing and translocating to nuclear to promote cell death. Besides, death of colon cancer cells could be significantly rescued by blocking PARP1 activity. These processes were inhibited by p53 deletion or mutation, while promoted by overexpression of SIRT6. Meanwhile, ROS production and protective autophagy were elevated after inhibition of AKT, might also play a role in the occurrence of parthanatos. Our findings demonstrated that targeting AKT inhibition induced the activation of parthanatos, which can be recognized as a novel potential therapeutic strategy for cancer.

## Supplementary Information

**Additional file 1: Fig. S1.** **a** After HCT116 cells were treated with 10 μM of SC66 and TIC10 for 24 h and 48 h respectively, Hoechst 33,258 staining was performed. **b** ROS production in HCT116 was tested by fluorescence microscope after treated with 10 μM SC66 for 12 h and Rosup was used as positive control. **c** ROS positive cells rates after treatment. **d** ROS production in HCT116 AKT knock down cells vs. HCT116 parental cells, tested by fluorescence microscope. **e** Quantitative analysis for AIF translocation. *p < 0.05, ***p < 0.001.

**Additional file 2: Fig. S2.** **a, b, c** dead cells were stained by trypan blue after SC66 (10 μM) treatment combined with or without Z-VAD (10 μM) and 3AB (10 μM) for 24 h (**a**). The combination treatment effect of Z-VAD (**b**) or 3AB (**c**) with SC66 were quantified. **d** Western blot analysis of PAR polymer induction by SC66 in RKO cells. **e** Puncta formation of GFP-LC3 in HCT116 after treated with 10 μM of SC66 and TIC10 for 24 h and 48 h respectively. **f, g** PAR polymer accumulation in HCT116 was detected by western blotting assay after administrated 10 μM SC66 for 24 h with or without autophagy inhibitors, 10 μM ULK101 (**f**) and 10 μM CQ (**g**).

**Additional file 3: Fig. S3.** **a** qRT-PCR assay tested the level of p53 after SC66 administration. **b** Exogenous co-immunoprecipitation was performed in HEK293T cells transfedted with YFP-p53. **c** co-immunoprecipitation with anti PARP1 antibody and IB with anti-p53 antibody was performed in HCT116 cells.

**Additional file 4: Fig. S4.** **a** The mutation type and rate of p53. **b** The mutation sites of p53. **c, d** The expression of PARP1 (**c**) and SIRT6 (**d**) in tumor tissue and normal tissue, according to online datasets.

## Data Availability

Not applicable.

## Abbreviations

AIF: Apoptosis-inducing factor
ATCC: American Type Culture Collection
co-IP: Co-Immunoprecipitation
CRC: Colorectal cancer
DCF: Dichlorofluorescein
EGFR: Epidermal growth factor receptor
FBS: Fetal bovine serum
FDA: Food and Drug Administration
HRP: Horseradish peroxidase
IF: Immunofluorescence
parthanatos: PARP1 dependent cell death
PARP1: Poly [ADP-Ribose] polymerase 1
PAR polymer: Poly(ADP-ribose) polymer
ROS: Reactive oxygen species
SPF: Specific pathogen-free
VEGFA: Vascular endothelial growth factor
