# Supplementary figures and images for: Inhibition of AKT induces p53/SIRT6/PARP1-dependent parthanatos to suppress tumor growth
Source: Cell Commun Signal. 2022 Jun 17;20:93. doi: 10.1186/s12964-022-00897-1 (PMC9205131; doi:10.1186/s12964-022-00897-1)

Supplemental Figure 1

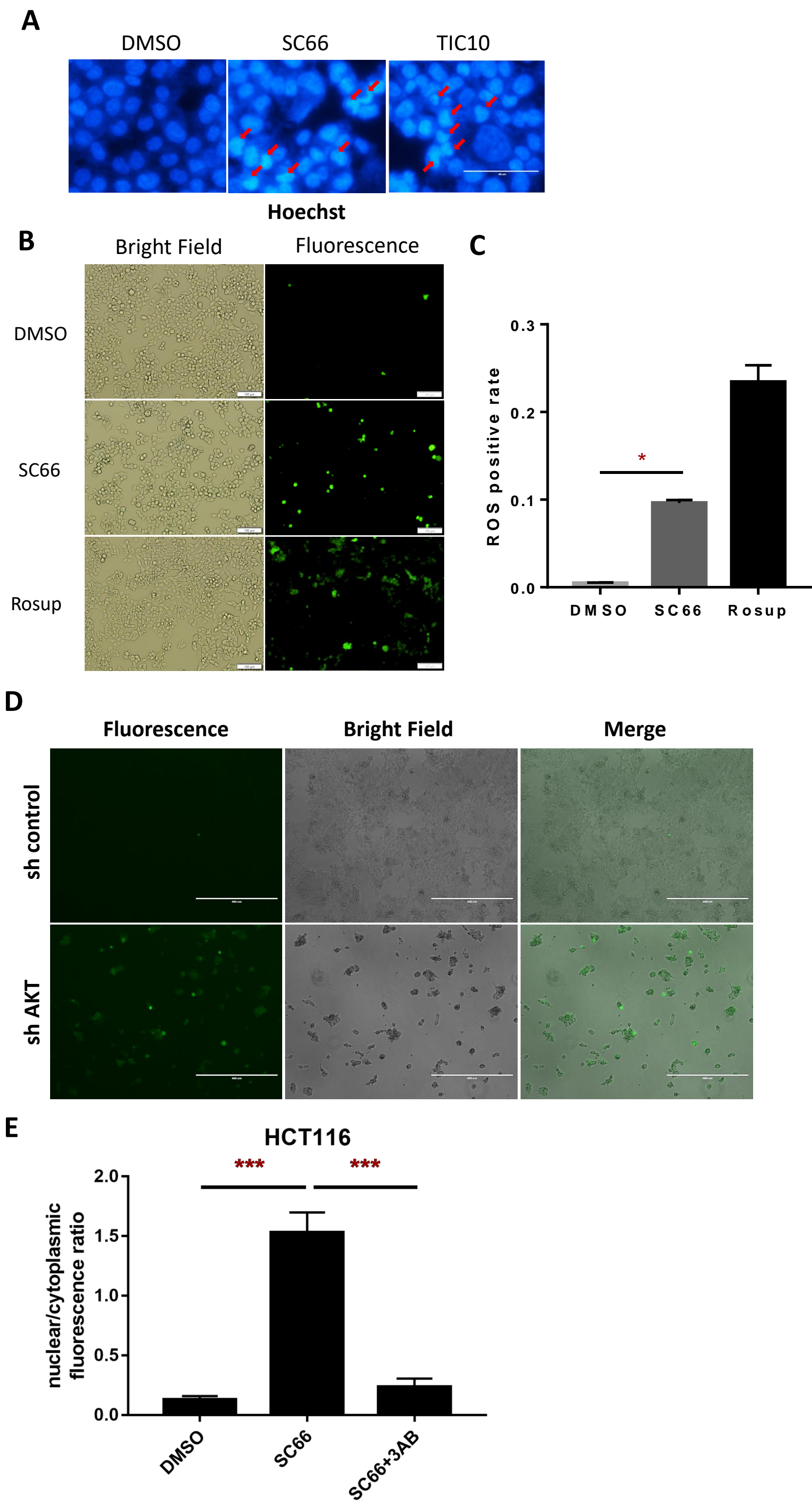

Supplement: Supplementary file 2 — Additional file 1: Fig. S1. a After HCT116 cells were treated with 10 μM of SC66 and TIC10 for 24 h and 48 h respectively, Hoechst 33,258 staining was performed. b ROS production in HCT116 was tested by fluorescence microscope after treated with 10 μM SC66 for 12 h and Rosup was used as positive control. c ROS positive cells rates after treatment. d ROS production in HCT116 AKT knock down cells vs. HCT116 parental cells, tested by fluorescence microscope. e Quantitative analysis for AIF translocation. *p < 0.05, ***p < 0.001. [file 12964_2022_897_MOESM2_ESM.pdf]

Supplemental Figure 2

A

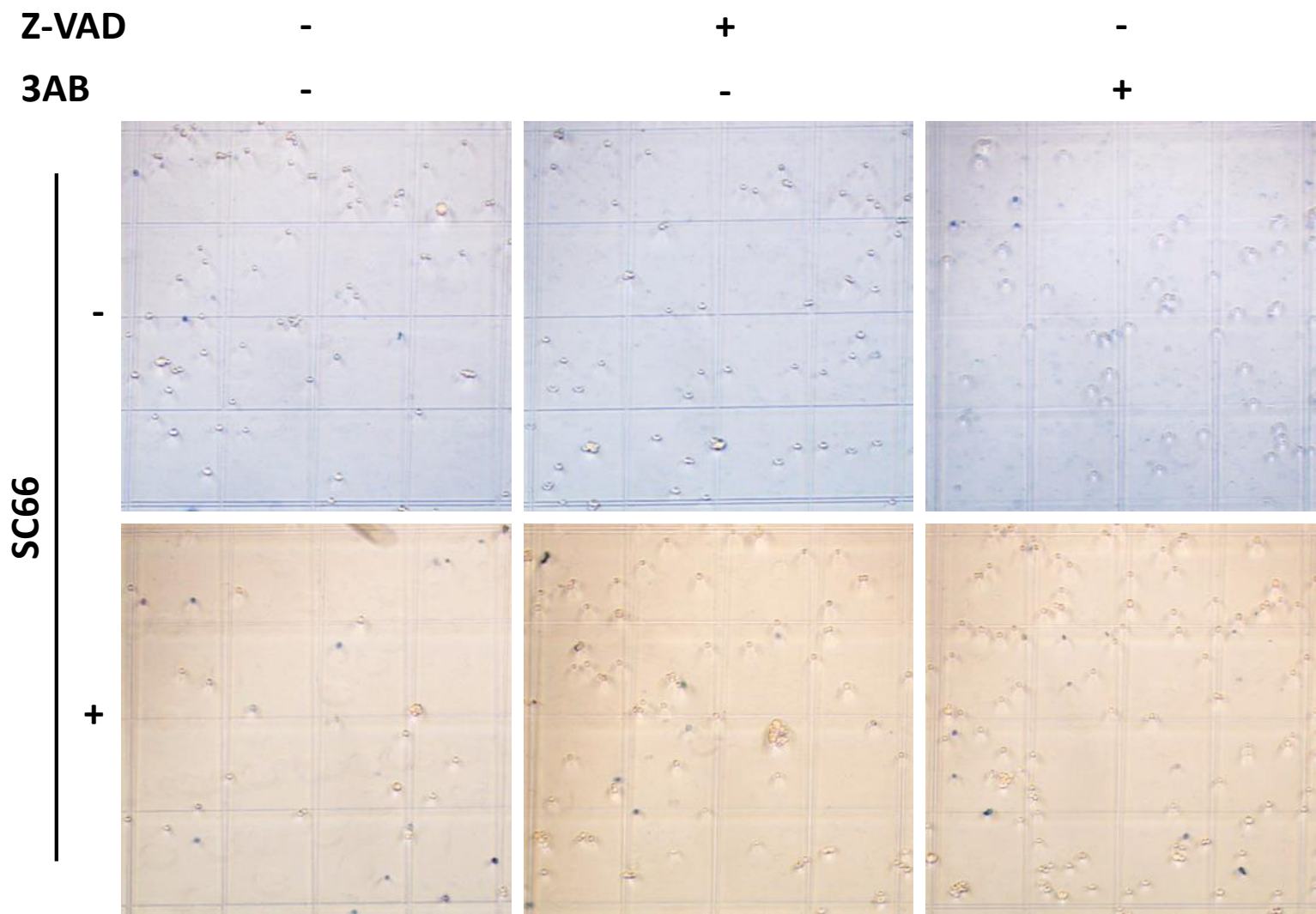

B

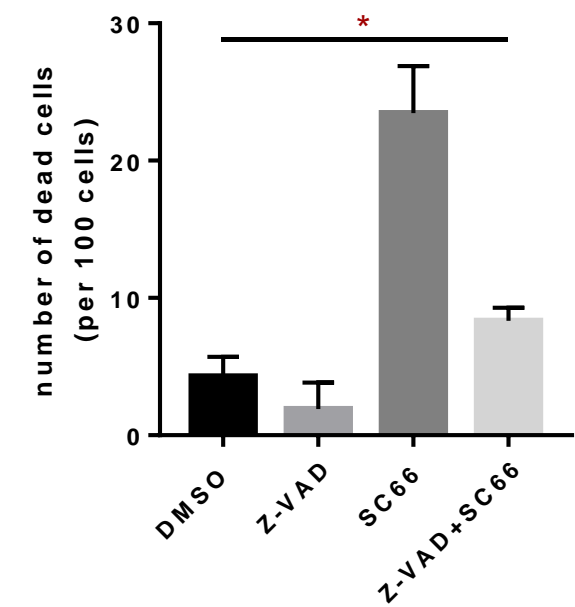

C

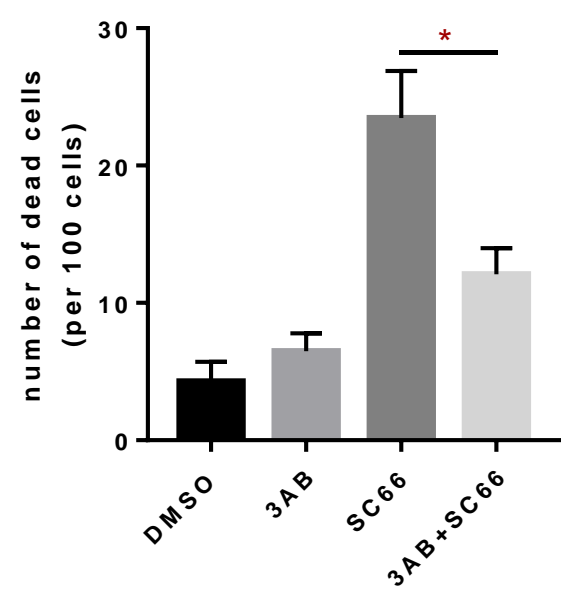

D

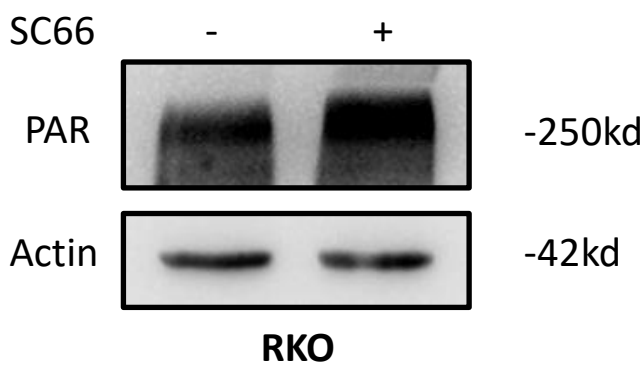

E

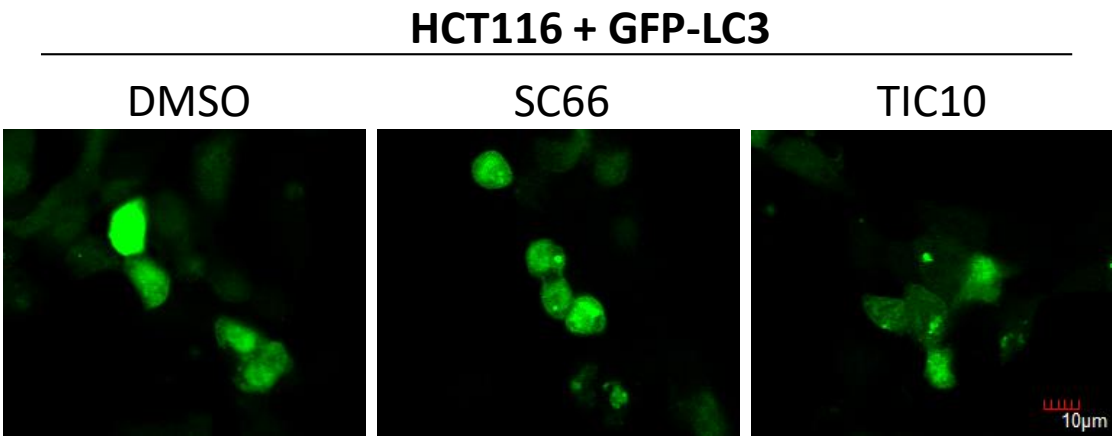

F

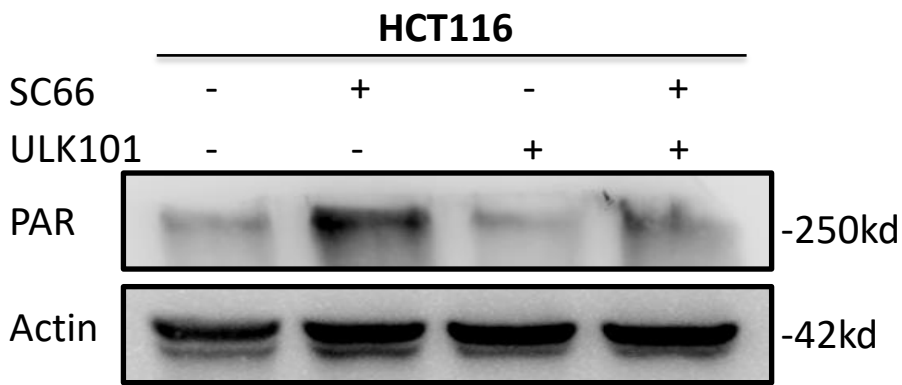

G

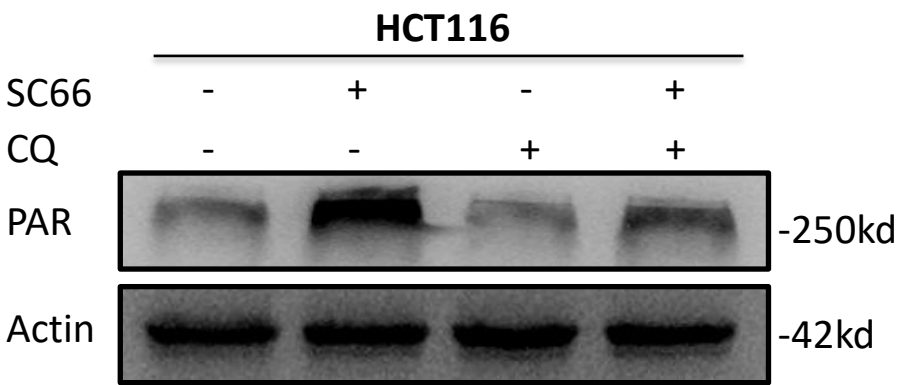

Supplement: Supplementary file 3 — Additional file 2: Fig. S2. a, b, c dead cells were stained by trypan blue after SC66 (10 μM) treatment combined with or without Z-VAD (10 μM) and 3AB (10 μM) for 24 h (a). The combination treatment effect of Z-VAD (b) or 3AB (c) with SC66 were quantified. d Western blot analysis of PAR polymer induction by SC66 in RKO cells. e Puncta formation of GFP-LC3 in HCT116 after treated with 10 μM of SC66 and TIC10 for 24 h and 48 h respectively. f, g PAR polymer accumulation in HCT116 was detected by western blotting assay after administrated 10 μM SC66 for 24 h with or without autophagy inhibitors, 10 μM ULK101 (f) and 10 μM CQ (g). [file 12964_2022_897_MOESM3_ESM.pdf]

Supplemental Figure 3

**A**

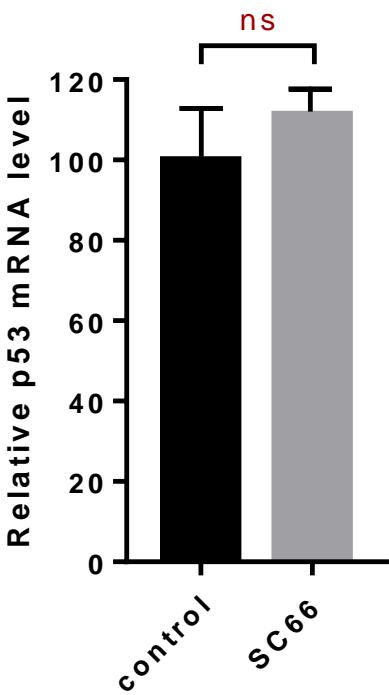

**B**

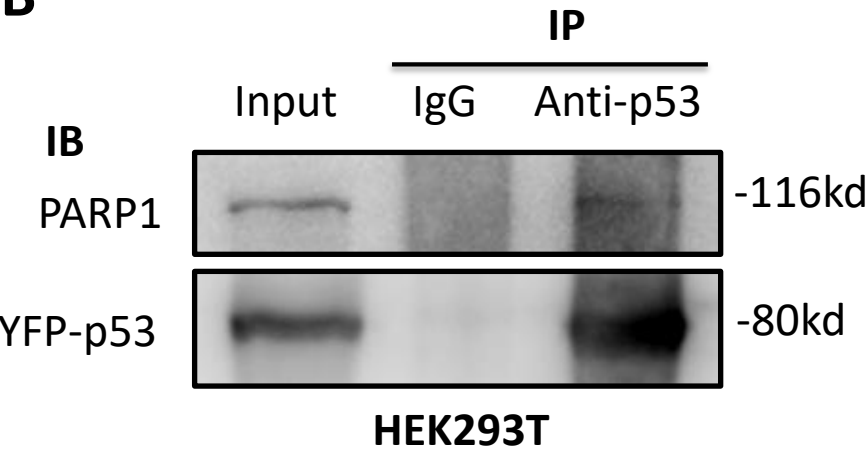

**C**

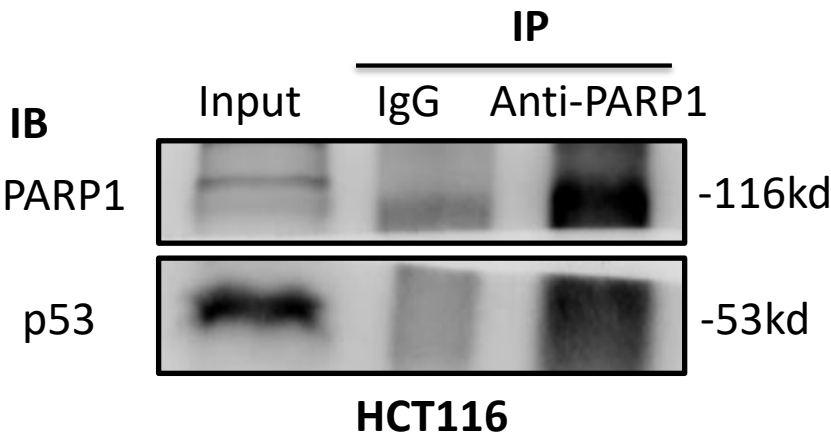

Supplement: Supplementary file 4 — Additional file 3: Fig. S3. a qRT-PCR assay tested the level of p53 after SC66 administration. b Exogenous co-immunoprecipitation was performed in HEK293T cells transfedted with YFP-p53. c co-immunoprecipitation with anti PARP1 antibody and IB with anti-p53 antibody was performed in HCT116 cells. [file 12964_2022_897_MOESM4_ESM.pdf]

# Supplemental Figure 4

A

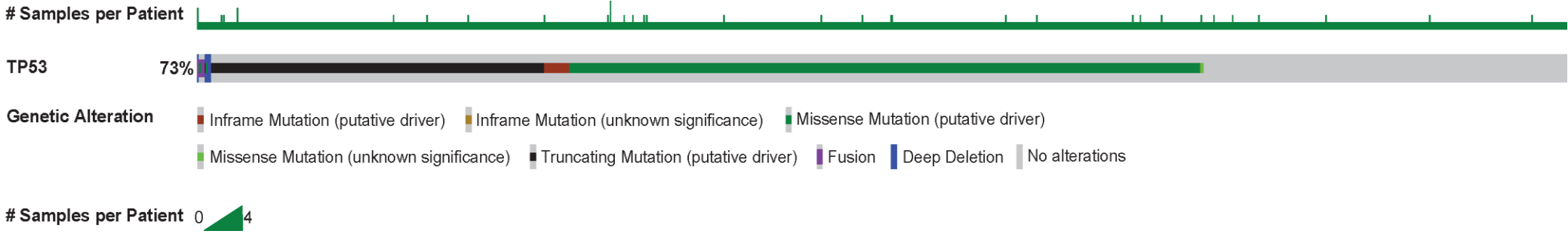

B

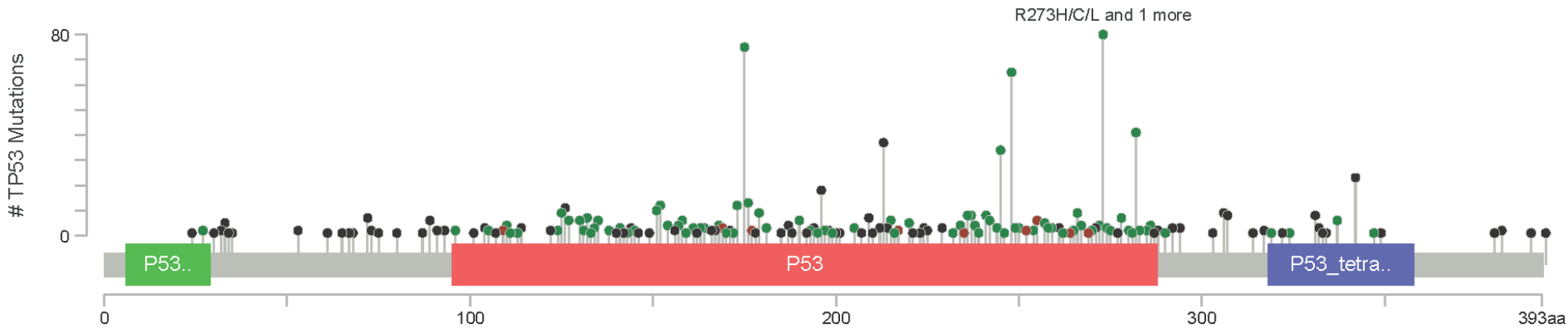

C

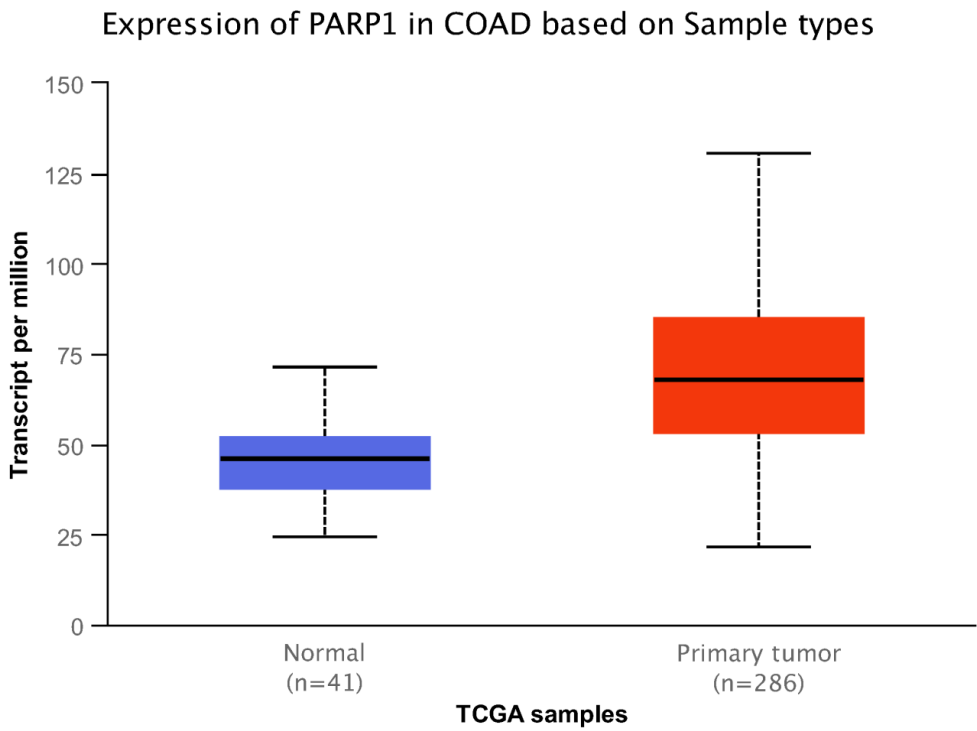

D

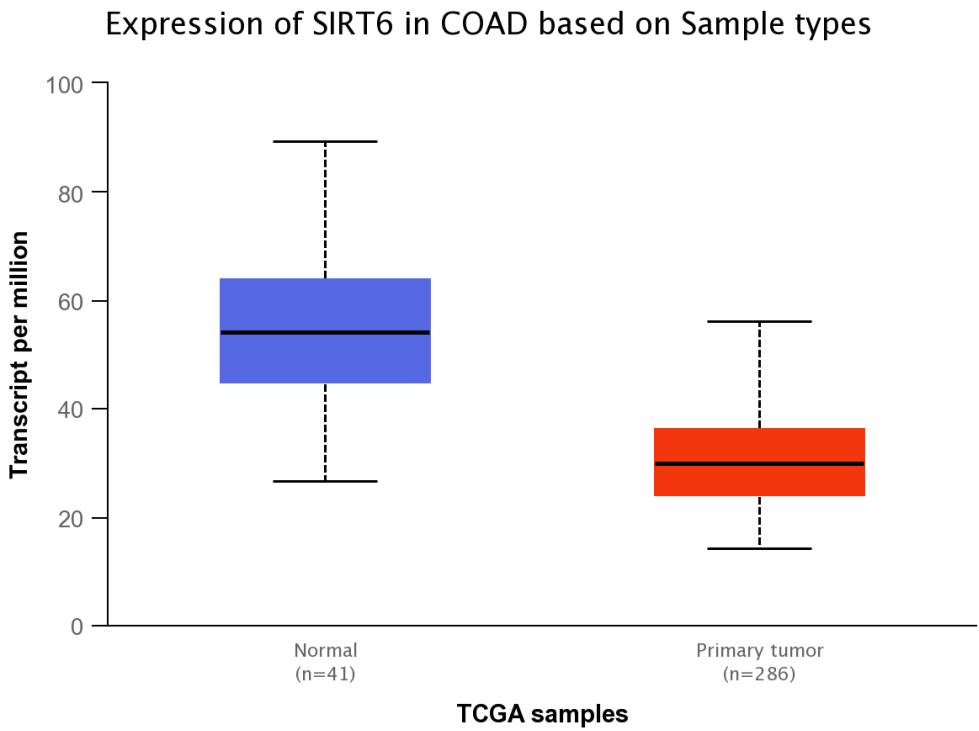

Supplement: Supplementary file 5 — Additional file 4: Fig. S4. a The mutation type and rate of p53. b The mutation sites of p53. c, d The expression of PARP1 (c) and SIRT6 (d) in tumor tissue and normal tissue, according to online datasets. [file 12964_2022_897_MOESM5_ESM.pdf]
